# Access to E.D. for mental disorders during the first pandemic wave of 2019 coronavirus epidemic (CoViD-19): Presentation and severity at a lombardy ED

**DOI:** 10.1192/j.eurpsy.2021.974

**Published:** 2021-08-13

**Authors:** G. Savioli, S. Pesenti, I. Ceresa, E. Oddone, M.A. Bressan

**Affiliations:** 1 Emergency Department, IRCCS Policlinico San Matteo, Pavia, Italy; 2 La Bonne Semence Social Cooperative Of Oltre Il Colle (bg) And In Cammino Social Cooperative Of San Pellegrino Terme (bg)., La Bonne Semence social cooperative of Oltre il Colle (BG) and In Cammino social cooperative of San Pellegrino Terme (BG)., Bergamo, Italy; 3 Department Of Public Health, University of Pavia, Pavia, Italy

**Keywords:** COVID-19 pandemic, Emergency department, metal disorder severity, Triage in mental desorder

## Abstract

**Introduction:**

The 2019 coronavirus epidemic (CoViD-19) in Italy originated in Lombardy, on February 21, 2020. The Fondazione IRCCS Policlinico San Matteo di Pavia has been involved in the management of the outbreak since its beginning. ED’ psychiatric population is considered fragile, at risk of under triage.

**Objectives:**

We evaluated all the population who went to the ED for mental disorder to assess the severity of cases evaluated as exit code and rate of hospitalization.

**Methods:**

We evaluated all patients accessing our ED for mental disorder from February 22 to May 1, 2020 and during the same period of the previous year.

**Results:**

We enrolled 345 patients. There was a severe reduction in the total number of accesses for mental disorder: 142 in the CoViD period and 203 in 2019. The vital parameters, age (mean about 40 years) and sex were overlapping without statistically significant differences. The priority codes for the medical examination were not different. CoViD pandemic patients have higher discharge severity codes (yellow and red) more frequently than in the reference period (9.9% vs 5.9%) and more frequently need hospitalization (25.3% vs 18.6%).

**Conclusions:**

The epidemic has led to a reduction of accesses for mental disorder. Patients had more frequent hospitalization needs and more severe exit codes. the data may be due to the fact that during the pandemic only the most serious patients access the E.D., but also to the fact that a pandemic has contributed to destabilizing this class of fragile patients.

